# Neurometabolic changes in neonates with congenital heart defects and their relation to neurodevelopmental outcome

**DOI:** 10.1038/s41390-022-02253-y

**Published:** 2022-08-22

**Authors:** Céline Steger, Maria Feldmann, Julia Borns, Cornelia Hagmann, Beatrice Latal, Ulrike Held, András Jakab, Ruth O’Gorman Tuura, Walter Knirsch

**Affiliations:** 1 Center for MR-Research, University Children’s Hospital, Zurich, Switzerland; 2 Pediatric Cardiology, Pediatric Heart Center, Department of Surgery, University Children’s Hospital, Zürich, Switzerland; 3 Children’s Research Center, University Children’s Hospital, Zürich, Switzerland; 4 Neuroscience Center Zürich, University of Zürich, Zürich, Switzerland; 5 University of Zurich, Zurich, Switzerland; 6 Child Development Center, University Children’s Hospital, Zurich, Switzerland; 7 Pediatric Cardiology, Inselspital Bern, Bern, Switzerland; 8 Department of Neonatology and Pediatric Intensive Care, University Children’s Hospital, Zurich, Switzerland; 9 Department of Epidemiology, Biostatistics and Prevention Institute UZH, Zürich, Switzerland

## Abstract

**Background:**

Altered neurometabolite ratios in neonates undergoing cardiac surgery for congenital heart defects (CHD) may serve as a biomarker for altered brain development and neurodevelopment (ND).

**Methods:**

We analyzed single voxel 3T PRESS H^1^-MRS data, acquired unilaterally in the left basal ganglia and white matter of 88 CHD neonates before and/or after neonatal cardiac surgery and 30 healthy controls. Metabolite ratios to Creatine (Cr) included glutamate (Glu/Cr), myo-Inositol (mI/Cr), glutamate and glutamine (Glx/Cr), and lactate (Lac/Cr). In addition, the developmental marker N-acetylaspartate to choline (NAA/Cho) was evaluated. All children underwent ND outcome testing using the Bayley Scales of Infant and Toddler Development Third Edition (BSID-III) at 1 year of age.

**Results:**

White matter NAA/Cho ratios were lower in CHD neonates compared to healthy controls (group beta estimate: −0.26, std. error 0.07, 95% CI: −0.40 – 0.13, *p* value <0.001, FDR corrected *p* value = 0.010). We found no correlation between pre- or postoperative white matter NAA/Cho with ND outcome while controlling for socioeconomic status and CHD diagnosis.

**Conclusion:**

Reduced white matter NAA/Cho in CHD neonates undergoing cardiac surgery may reflect a delay in brain maturation. Further long-term MRS studies are needed to improve our understanding of the clinical impact of altered metabolites on brain development and outcome.

**Impact:**

- NAA/Cho was reduced in the white matter, but not the gray matter of CHD neonates compared to healthy controls.
- No correlation to the 1-year neurodevelopmental outcome (Bayley-III) was found.
- While the rapid change of NAA/Cho with age might make it a sensitive marker for a delay in brain maturation, the relationship to neurodevelopmental outcome requires further investigation.

## Introduction

Congenital heart defects (CHD) are among the most common birth defects, with one in a hundred newborns affected.^1,2^ Mild to moderate impairments in different areas of neurodevelopment (ND), including motor and cognitive domains, are frequently observed among children with complex CHD.^3,4^ While ND impairments arise due to a combination of patient-specific internal (e.g., genetics, diagnosis) and external factors, they are thought to be related to alterations in brain development.

Studies using magnetic resonance (MR) imaging have revealed various patterns of altered brain development in patients with CHD, such as total and regional brain volume,^5,6^ and delayed brain maturation.^7,8^ In addition, an increased prevalence of white matter injuries (WMI) has been described.^9,10^ Importantly, these neuroimaging findings could be linked to the ND outcome in these patients.^11–15^ However, even in the absence of structural abnormalities, brain metabolism can be altered due to a reduction of cerebral oxygen delivery and consumption as well as altered cerebral perfusion associated with the specific type of CHD.^16^ This may lead to more subtle changes on the metabolic level of neurons and glial cells potentially affecting ND outcome.

Metabolic compounds can be studied by magnetic resonance spectroscopy (MRS) and may serve as a biomarker reflecting different aspects of cellular functions. For instance, N-acetylaspartate (NAA), serves as a marker for neuronal function and neuronal density, myo-Inositol (mI) for glia and intracellular signal transduction (second messenger), choline-containing compounds (Cho) reflect cell membrane content or membrane turnover, glutamate (Glu) is a marker for neurotransmitter activity, glutamate and glutamine (Glx) and creatine containing compounds (Cr) reflect metabolic activity, while lactate may serve as an indicator for hypoxia.^17^

During the fetal and neonatal period, neurometabolic concentrations undergo substantial physiological changes, reflecting brain development and its underlying cellular processes. For example, the intracerebral concentrations of Cr and NAA increase, while concentrations of Cho and mI decrease.^18–20^ Due to the rapid increase in NAA and the corresponding decrease in Cho with development,^21,22^ the ratio of NAA/Cho has been suggested to represent a marker for brain maturation, sensitive both to changes in neuronal development (NAA) and cell membrane turnover and myelination (Cho).^23^ In neonates with severe CHD, studies with a focus on neurometabolic changes found decreased NAA/Cho^24,25^ and increased Lac/Cho ratios relative to values seen in healthy neonates.^25^

However, these studies did not report on the relationship between the metabolic alterations and ND outcome, something that has been investigated in preterm infants.^26^ Preterm infants are a patient population with a similar risk profile for impaired ND and neurometabolites measured at term equivalent age might be associated with outcome in this population.^27^ Alterations in neurometabolic concentrations and ratios determined by MRS may therefore serve as biomarkers for delayed brain development in children at risk for impaired ND, allowing for tailored timely intervention to improve patient-individual outcome.

In our study, we hypothesized that neurometabolite ratios in CHD neonates before and after neonatal cardiac surgery are altered in comparison to those of healthy controls and may be associated with impaired ND outcome at 1 year of age determined by the Bayley Scales of Infant and Toddler Development, Third Edition (BSID-III). Therefore, the aims of the study were twofold: (1) to identify altered neurometabolites in the CHD group compared to a healthy control group and (2) to investigate the relationship between altered neurometabolite levels and the Bayley composite scores (BCS) at 1 year of age.

## Methods

### Subjects

Data were collected in a single-center prospective cohort study of the Research Group Heart and Brain.^12,28^ The cantonal ethical committee approved the study (KEK StV-23/619/04), and parents or legal guardians provided written informed consent. Between December 2009 and April 2020, 125 term newborns (>36 weeks of gestation) with CHD scheduled for neonatal cardiac surgery were enrolled in the study. Neonates with suspected or confirmed genetic disorder, born preterm (before the 36th week of gestation), who did not undergo surgery during the neonatal period (first 6 weeks of life) or with no available written consent were excluded. Patients underwent cerebral MRI before and/or after cardiac surgery. In addition to the patient population, 52 healthy term newborns from the well-baby maternity unit of the University Hospital Zurich were recruited between 2011 and 2016 as healthy controls. These healthy controls were scanned once. A total of 88 CHD and 30 controls had MRS data available and underwent a BSID-III assessment at 1 year of age. A more detailed overview of the dataset is given in Supplementary Fig. 1. As MRS was a secondary outcome of the overall study, the sample size was not formally calculated prior to this analysis, but was based on the availability of data.

### Magnetic resonance spectroscopy

**Fig. 1:**
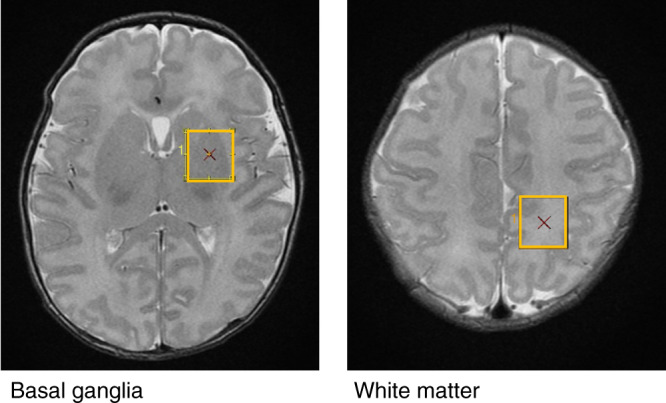
MRS voxel locations within the basal ganglia and white matter.

MR data were acquired on a 3.0T scanner (GE Healthcare, Milwaukee, WI) using an eight-channel head coil. During the study, a scanner upgrade from the HD.xt to the MR750 platform was performed. Neonates were scanned during natural sleep. Earplugs and Minimuffs were used for noise protection. Patients were in a stable hemodynamic state at the time of MRI, as described previously.^6,12^ Short-echo time (TE) H^1^-proton MRS was acquired in two voxels placed in the basal ganglia/thalami (16 × 16 × 16 mm^3^) and white matter (16 × 16 × 16 mm^3^) (see Fig. 1 for voxel positions). A single voxel Point RESolved Spectroscopy (PRESS) sequence (repetition time = 3000 ms, TE = 35 ms, 96 averages) was used for the acquisition. Spectra were quantified using LCModel, a fully automated spectral fitting software package that estimates the concentration of the metabolites, uncertainty, and the concentration ratio to creatine (Cr) of each metabolite. In LCModel uncertainties are calculated as the percentages of the Cramer–Rao lower bound (CRLB) of the metabolite fit (% SD). The following metabolite ratios to Creatine were analyzed: glutamate (Glu/Cr), glycerophosphorylcholine with phosphorylcholine (Cho/Cr), myo-Inositol (mI/Cr), glutamate and glutamine (Glx/Cr), lactate (Lac/Cr). N-acetylaspartate/Cho (NAA/Cho) was additionally calculated from the measured concentrations of NAA and Cho.

Collected spectra underwent visual quality control for fitting errors, and spectra where the relative CRLB for NAA or Cr exceeded 10% were excluded.

### Neurodevelopmental outcome

CHD patients and healthy controls underwent neurodevelopmental outcome testing at 1 year of age using the BSID-III. BSID-III provides composite scores for three domains, namely the motor composite score (MCS), the language composite score (LCS), and the cognitive composite score (CCS). BCS are age adjusted and were designed to have a mean score of 100 and a standard deviation of ±15. The assessment was administered by trained developmental pediatricians, who were not blinded to clinical and major structural MRI findings.

### Cardiac diagnosis and surgical procedure

Cardiac diagnoses for the CHD participants were divided into subgroups including d-Transposition of the Great Arteries (dTGA), left ventricular outflow tract obstructions (LVOTO), right ventricular outflow tract obstructions (RVOTO), single ventricle physiology (SVP), and other.

Cardiac surgery included biventricular repair by arterial switch or Rastelli operation for patients with dTGA, complex aortic arch surgery, systemic-pulmonary shunt procedure, and neonatal Fallot repair. For univentricular palliation, Norwood-type stage I palliation for patients with hypoplastic left heart syndrome and other forms of univentricular physiology with a hypoplastic aortic arch was performed. In the case of aortic arch surgery, i.e., Norwood repair, cardiopulmonary bypass (CPB) surgery was performed under moderate hypothermia (25 °C) with selective regional cerebral perfusion. Modified ultrafiltration was used at the end of CPB surgery. Postoperative intensive care and further follow-up were evaluated until the end of the first year of life.

### Variables

The dependent variable of the first research question was the metabolite ratios: NAA/Cho, mI/Cr, Glu/Cr, Glx/Cr, and Lac/Cr. The variable scanner software was introduced to account for whether participants were scanned before or after the MR scanner upgrade. Gestational age at scan is a known predictor for some metabolites and sex is a potential confounder^29,30^ and thus were included in the analyses. The dependent variable of the second research question was ND outcome (CCS, LCS, and MCS) at 1 year. Due to its known correlation with neurodevelopmental outcome,^31^ the socioeconomic status (SES, ranging from 2 to 12) was estimated based on a sum score of maternal education and paternal occupation (each 1–6) and included as a covariate. To account for the diagnosis as a covariate for outcome, a binary diagnosis variable dTGA and non-dTGA was introduced. This binarization was done to keep variables in the model limited. Patients with dTGA undergoing arterial switch were used as the most homogenous CHD group and compared with other type of CHD, termed as non-dTGA group, which included a more heterogenous group of CHD. In additional exploratory analyses, the classification of moderate and severe types of CHD was used^32^ and the covariate WMI at scan (yes/no) was added.

### Statistical analysis

Statistical analysis was carried out in R (R version 4.0.4).^33^ Descriptive statistics for both groups are shown as mean and standard deviation, median and interquartile range, or number and percentages. Exploratory group comparisons were performed using a *t*-test, Wilcoxon rank-sum test, or *χ*^2^ test, as appropriate. The metabolite ratios are presented for healthy controls, CHD preoperative, and CHD postoperative as mean and standard deviation and median and interquartile range. The percentage of missing values for each metabolite is reported. Lac/Cr ratio was not normally distributed and was close to zero. The data were therefore logarithmically transformed after the addition of 0.001 to each value to avoid log transformation of zero values.

To investigate differences in metabolite ratios between the CHD and the control group, mixed effect models were employed (“lme4” package R). Models consisted of the metabolite ratio as the dependent variable and group (CHD or healthy control), centered gestational age at scan, sex, and scanner software as independent variables. A random intercept for each subject was introduced to account for the repeated measurements in subjects of the CHD group, as the models' combined metabolite ratios from pre- and post-surgery scans for patients. Linear models without random effects were also fitted to qualitatively confirm the robustness of the estimated models (data not shown). Normality of residuals was checked by visual inspection of histograms, Residuals vs Fitted, and QQ plots. *P* values of the mixed-effects models were obtained using the “lmertest” package in R. To account for multiple comparisons, *p* values were adjusted according to the Benjamini–Hochberg procedure,^34^ using the “stats” package in R. *P* values <0.05 were considered statistically significant.

In addition, a post hoc analysis was carried out on the second cohort only to explore the difference between groups in the absence of the scanner upgrade confounder.

The correlation between white matter NAA/Cho and the neurodevelopmental outcome was tested in the CHD group only, using linear models. Separate models were generated for pre- and postoperative metabolite data. The dependent variable of each linear model was one of the three BSID-III composite scores, while independent variables gestational age at MRI, metabolite ratio, SES, and diagnosis (dTGA vs non-dTGA or severe vs moderate) were chosen. In addition, models correcting for the presence of WMI at scan timepoint were explored. Normality of residuals was checked by visual inspection of residuals histograms, Residuals vs Fitted, and QQ plots. Two-sided *p* values <0.05 were considered significant.

In a post hoc analysis, we explored whether patients with WMI had lower white matter NAA/Cho than patients without.

The study was reported according to STROBE guidelines.

## Results

### Study population

**Fig. 2:**
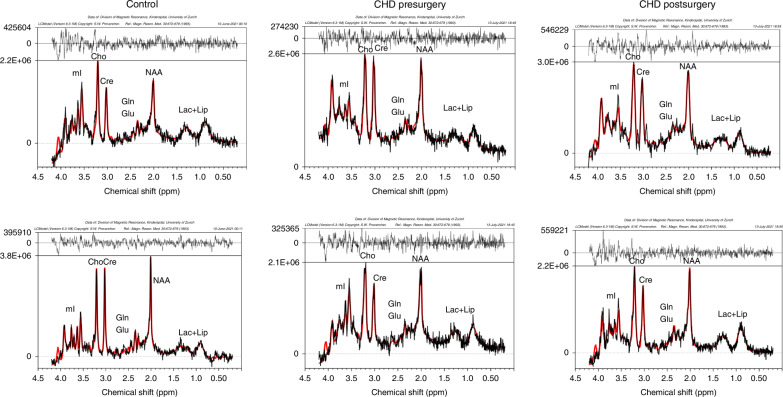
Representative spectra of control and patient pre- and postoperatively. Basal ganglia spectra are depicted in the top row, while white matter spectra are shown in the bottom row. The chemical shift (ppm) is shown on the X axis, and the spectral data are shown in black with the LCModel fit overlaid in red. The residuals between the data and the fit are shown above each spectrum. NAA N-acetylaspartate, Cho choline, Cre creatine, mI myo-Inositol, Glx glutamate and glutamine, Glu glutamate, Lac lactate, Lip lipid.

Demographic characteristics and other variables included in the study of CHD patients and healthy controls are given in Table 1. In the CHD group, 62 preoperative spectra and 67 postoperative spectra were available (out of the 88 CHD subjects, 41 CHD cases had two MRS spectra, 21 had preoperative spectra only, and 26 had postoperative spectra only) (Fig. 2). The most frequent diagnosis was dTGA (56.8%) and 61.9% of CHD patients underwent CPB surgery between the two MR timepoints. Eleven CHD patients had white matter lesions in the preoperative scan and ten patients had white matter lesions in the postoperative scan. For further details see Supplementary Table 1. CHD patients had an SES of 9 IQR [7, 10] (median [IQR]) that was lower than that of the control group who had an SES of 12 IQR [11, 12] (*p* = 0.001). A total of 63 CHD (71.6%) and 20 (66.7%) control patients were scanned after the scanner upgrade (*χ*^2^ test: *p* value: 0.781).

**Table 1:** Baseline characteristics.

	CHD	Healthy controls	*p* value^a^
Number (*n*)	88	30	
Male sex	63 (71.6%)	15 (50.0%)	0.046* (c)
Gestational age (weeks)	39.32 (1.24)	39.63 (1.24)	0.245 (a)
Birth weight (kg)	3.33 (0.45)	3.37 (0.41)	0.460 (a)
Diagnosis	88 (100%)		
dTGA (*n*, %)	50 (56.8)	–	–
LVOTO (*n*, %)	9 (10.2)	–	–
RVOTO (*n*, %)	9 (10.2)	–	–
SVP (*n*, %)	11 (12.5)	–	–
Other^b^ (*n*, %)	9 (10.2)	–	–
Cardiopulmonary bypass (*n*, %)	73 (61.9)	–	–
Socioeconomic status	8 [7–10]	12 [11–12]	<0.001* (b)

Characteristic variables stratified by group. Categorical and binominal variables presented as number and percentage (*n* (%)). Mean and standard deviation (m (SD)) or median and interquartile range (m [IQR]) for continuous variables. The variable scanner software was a binominal categorical variable introduced to account for a scanner upgrade during the study.

*D-TGA* d-Transposition of the Great Arteries, *LVOTO* left ventricular outflow tract obstruction, *RVOTO* right ventricular outflow tract obstruction, *SVP* single ventricle physiology.

^a^Exploratory *p* values show uncorrected *p* values, asterisks (*) indicate *p* value <0.05, the used test is specified in brackets as follows:

(a) *t*-test, (b) Wilcoxon rank-sum test, and (c) *χ*^2^ test.

^b^Other included: ventricular septal defect, L-TGA, truncus arteriosus communis, and total anomalous pulmonary venous connection.

### Metabolite ratio alterations

**Fig. 3:**
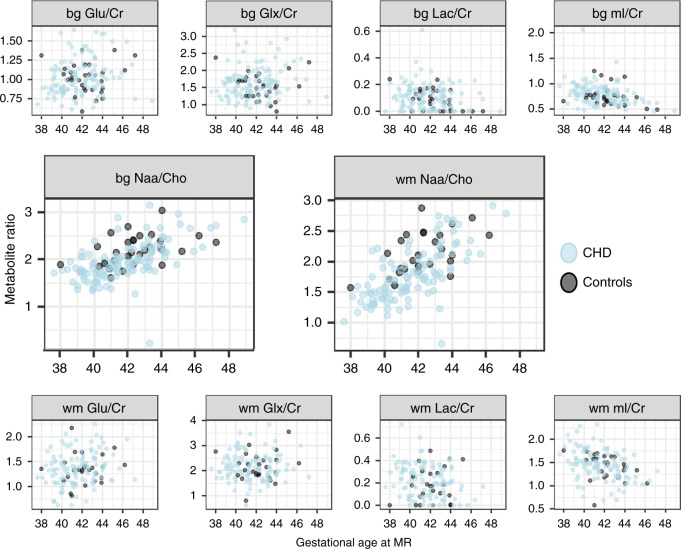
Metabolite ratios versus gestational age. X axis: gestational age in weeks, Y axis: metabolite ratio. First row = white matter (wm), third row = basal ganglia (bg), middle row = NAA/Cho in wm and bg. datapoints: blue = CHD cohort, black = healthy controls.

**Fig. 4:**
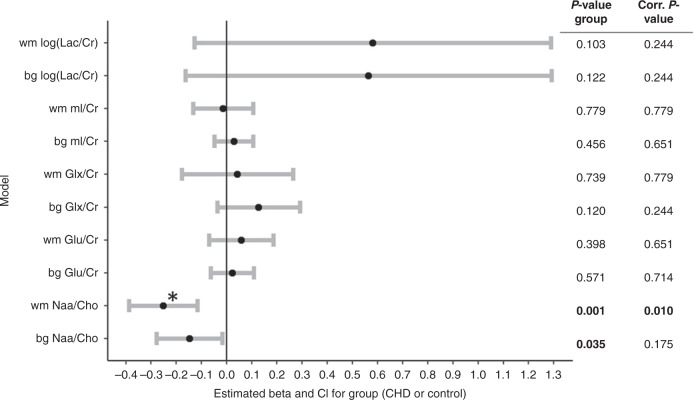
Metabolite differences between groups: estimated betas and confidence intervals. For each model, the estimated beta coefficient of the group, its 95% confidence interval (CI), and *p* values are listed. Complete models are described in Supplementary Table 3. The figure to the right illustrates the results as follows: A beta coefficient for the group that is positive describes a larger metabolite ratio for the CHD group. CI crossing 0.0 implies that there is no significant effect. Lactate was logarithmically transformed, for results see Supplementary Table 2. Asterisk (*) indicates the results that remained significant after false discovery rate correction. NAA N-acetylaspartate, Cho choline, Cr creatine, mI myo-Inositol, Glx glutamate+glutamine, Glu glutamate, Lac lactate.

An increase of Naa/Cho with gestational age can be observed in both the white matter and the basal ganglia, while mI/Cr decreases with gestational age. Figure 3 illustrates the relationship of each metabolite ratio with the gestational age at the time of measurement. Table 2 shows the measured cerebral metabolites for healthy controls and CHD neonates before and after cardiac surgery. More data were missing for the white matter measurement, possibly due to the order of the spectra in the scanning protocol, in which the basal ganglia spectrum is acquired before the white matter spectrum. For the groupwise analysis in the full cohort, the beta coefficients of the group for each metabolite and location are reported in Fig. 4. The main effect of group was significant for NAA/Cho in the white matter. Belonging to the CHD group was associated with a 12.3% reduction in NAA/Cho ratio in the white matter (beta estimate: −0.26, std. error 0.07, 95% CI: −0.40 to 0.13, *p* value <0.001, FDR corrected *p* value = 0.010). NAA/Cho ratio in the basal ganglia was not significant after correction for multiple testing (beta estimate: −0.14, std.error: 0.07, 95% CI: −0.27 to −0.01, *p* value = 0.035, FDR corrected *p* value = 0.175). A positive relationship with gestational age at scan was modeled in both locations for NAA/Cho and for Glu/Cr, while a negative relationship was described in both locations for mI/Cr and in the basal ganglia for log (Lac/Cr). The analysis on only the second cohort (after scanner software upgrade) had less power, but reproduced the main finding of a decrease in NAA/Cho within the white matter in the CHD group. A detailed result table of each model is provided in Supplementary Tables 2 and 3. The necessity of an interaction term of gestational age at scan and group (CHD vs control) was explored, but the interaction was not significant and therefore not included in the models (data not shown).

**Table 2:** Metabolite ratios.

	Healthy controls	CHD pre-surgery	CHD post-surgery	Missing data
*n*	30	62	67	
Gestational age at scan	42.5 (1.9)	40.2 (1.4)	43.0 (2.0)	0
Basal ganglia				
NAA/Cho	2.2 (0.31)	1.76 (0.21)	2.13 (0.44)	2
Cho/Cr	0.36 (0.04)	0.39 (0.04)	0.37 (0.07)	2
NAA/Cr	0.78 (0.1)	0.69 (0.07)	0.79 (0.12)	2
mI/Cr	0.75 (0.19)	0.82 (0.15)	0.77 (0.24)	2
Glu/Cr	1.02 (0.19)	0.97 (0.19)	1.08 (0.25)	2
Glx/Cr	1.53 (0.37)	1.56 (0.38)	1.71 (0.49)	2
Lac/Cr	0.09 [0.00, 0.15]	0.11 [0.04, 0.17]	0.11 [0.04, 0.15]	2
White matter				
NAA/Cho	2.14 (0.35)	1.57 (0.26)	2.00 (0.45)	17
Cho/Cr	0.49 (0.07)	0.54 (0.07)	0.48 (0.08)	17
NAA/Cr	1.04 (0.15)	0.83 (0.11)	0.96 (0.16)	17
mI/Cr	1.4 (0.27)	1.55 (0.29)	1.35 (0.31)	17
Glu/Cr	1.35 (0.29)	1.31 (0.32)	1.40 (0.32)	17
Glx/Cr	2.15 (0.55)	2.18 (0.52)	2.12 (0.53)	17
Lac/Cr	0.17 [0.08, 0.29]	0.24 [0.14, 0.33]	0.19 [0.07, 0.32]	17

Stratified by group and timepoint of measurement. Reported as mean and standard deviation (m (SD)). Lac/Cr is reported as median and interquartile range (m [IQR]) as it was nonnormally distributed. Missing data show the percentage of data missing in the dataset that was imputed.

*NAA* N-acetylaspartate, *Cho* choline, *Cr* creatine, *mI* myo-Inositol, *Glx* glutamate and glutamine, *Glu* glutamate, *Lac* lactate.

In an additional post hoc exploratory analysis we found no evidence that patients with WMI had different white matter NAA/Cho ratios than patients without WMI (data not shown).

### Neurodevelopmental outcome

Overall, the healthy controls and the CHD patients had a good outcome. BCS were found to be reduced in the CHD group, particularly for the cognitive and motor domains (exploratory linear model *p* values: CCS *p* = 0.04, MCS *p* = 0.01, LCS *p* = 0.57) (Table 3).

**Table 3:** Bayley composite scores.

Score	Healthy controls	CHD	*p* value	Missing
CCS	116 (11.92)	104.77 (14.12)	0.04* (a)	0
LCS	98.60 (10.85)	94.07 (12.48)	0.57 (a)	2
MCS	104.70 (9.77)	92.23 (15.17)	0.01* (a)	0

Determined at 1 year of age in patients with CHD and healthy controls. Missing data show the percentage of data missing in the dataset. Composite scores from Bayley Scales of Infant and Toddler Development, Third Edition (BSID-III).

*CCS* Bayley cognition composite score, *LCS* Bayley language composite score, *MCS* Bayley motor composite score.

Exploratory *p* values show uncorrected *p* values, asterisks (*) indicate *p* value <0.05, the used test is specified in brackets as follows:

(a) *p* values of group variable in linear model including socioeconomic status as covariate.

### Correlation between metabolite ratio and neurodevelopmental outcome

There was no evidence for a correlation between metabolite ratio (white matter NAA/Cho) and BCS (Supplementary Tables 4–7) in the explored models. The categorization of moderate vs severe did lead to higher *R*^2^ in the models compared to non-TGA vs TGA categorization. The highest *R*^2^ of 0.34 (adjusted *R*^2^ 0.27) was found for the linear model with dependent variable MCS and independent variables preoperative NAA/Cho, diagnosis (moderate vs severe), age at MRI, and SES. Severe CHD was associated with a reduced, while higher SES was associated with an increased motor score both pre- and postoperatively. As expected SES was associated with outcome in most of our explored models. WMI in postoperative scans was associated with language outcome scores, but not other scores. As the correlation of interest (NAA/Cho and outcome) was not significant in any of the models, no correction for multiple testing was performed.

## Discussion

In this study, we analyzed H^1^-MRS spectra of CHD neonates and healthy controls to evaluate metabolic alterations in CHD patients undergoing early neonatal cardiac surgery. We found significantly reduced NAA/Cho ratios for the CHD group compared to healthy controls in the white matter, but not the basal ganglia. The remaining cerebral metabolite ratios such as Lac/Cr, Glx/Cr, Glu/Cr, and mI/Cr were comparable between the CHD patients and healthy controls. Furthermore, we could confirm the physiological age-related rapid changes within the first month of life including a significant increase in NAA/Cho. We found no evidence for a correlation between white matter NAA/Cho and ND outcome at 1 year of age.

### Age-related and groupwise differences in MRS metabolite ratios

Age-related changes were evident in both CHD patients and healthy controls. Our data showed a rapid increase in NAA/Cho with age, while mI/Cr ratios declined with gestational age, consistent with findings from previous studies.^18,20^

The main alteration observed in the CHD was a 12.3% reduction in NAA/Cho ratios in the white matter group compared to the healthy controls. This observation supports previous reports of a 10% overall reduction of NAA/Cho ratios in neonates with CHD.^25^ A cross-sectional study in fetuses discussed the possibility of progressively lower NAA/Cho during the third trimester in CHD patients compared to healthy controls.^35^ We explored the interaction between the group and gestational age at scan, which was non-significant and therefore not included in our model. By using a linear model for our analysis, we assume and limit ourselves to a linear relationship between gestational age and metabolite ratios. Given the rapid changes with age, the inter- and intra-subject variability (reproducibility of MRS measurement),^36,37^ large longitudinal comparative studies are required to further investigate whether metabolites change at a similar rate over time in healthy controls and CHD patients.

Since our study design included voxels in the gray matter (basal ganglia) as well as the cerebral white matter, we also investigated the regional specificity of altered metabolite ratios in CHD. While NAA/Cho levels were reduced in the white matter, alterations in the basal ganglia were not significant, both with and without imputation for missing data. Metabolite ratios might therefore be altered in a regionally specific manner. NAA and Cho are thought to reflect neural integrity and brain development during the neonatal period, with NAA increasing^38^ and Cho decreasing with age.^20^ Cho plays a role in membrane turnover,^17,39^ while NAA is present in neurons and oligodendrocytes and could serve as a metabolite trafficking system supporting oligodendrocyte metabolism during brain development and in response to brain injury.^40^ While patients with CHD are at risk for brain injuries,^10^ a recent multi-center study showed that WMI seem to occur in a characteristic distribution pattern, due to the regional differences in brain maturation.^9^ The vulnerability of the oligodendrocyte progenitor cells that are present in the neonatal CHD brain due to delayed maturation is a proposed pathological mechanism underlying WMI.^41,42^ In addition, decreased NAA/Cho ratios have previously been associated with preoperative brain injuries in this population.^43^ This indicates that the location of MRS acquisition might be of importance when trying to establish a relation to outcome, and MRS data from the white matter might be more sensitive to pathological changes associated with CHD.

In our cohort, we only had a few patients with WMI, and we could not find evidence that this was associated with lower white matter NAA/Cho compared to patients without WMI. However, this post hoc analysis was exploratory and has to be interpreted with care, given the low frequency of WMI in our cohort. We found no evidence for groupwise alterations in any of the other metabolite ratios, although previous studies reported increased levels of lactate^25^ in the brains of CHD children, and lactate levels are also increased in neonates suffering from hypoxia or ischemic injuries.^44^

### BSID-III outcome after 1 year

The overall good outcome in CHD patients is likely to be the result of a variety of factors, as ND in itself is multifactorial. In our cohort, we report a high SES, a factor known to impact ND, and low WMI, which potentially can impact ND negatively. As in this MR study, only hemodynamically stable neonates could be scanned, and critically ill patients might be missing, which is not fully representative of the clinical population. This may have contributed to an overall low rate of WMI and overall good outcome.

### Altered cerebral metabolites at neonatal age and BSID-III outcome after 1 year

Few studies have investigated the relationship between cerebral metabolites and BSID in CHD children. In a previous study Park et al.^45^ reported a potential link between altered metabolite ratios measured at 1 year and BSID-II scores of a dTGA patient cohort, suggesting that neurodevelopmental delay may reflect consequences of altered metabolism. Importantly, they correlated MRS findings with ND both obtained at an age of 1 year, while we aimed to examine the predictive value of altered perioperative MRS on the subsequent outcome. With this approach, we found no evidence for a correlation between NAA/Cho ratios and ND outcome at 1 year of age. Serial MRS data may provide important insight into whether children with CHD demonstrate an ongoing impaired brain metabolism during the first year of life or a “normalization” of brain metabolism until the end of the first year of life. Furthermore, the sensitivity of the used Bayley scales (II or III generation) and the long-term effect of altered metabolism on ND until school age has to be determined.

More data have been reported on the relationship between cerebral metabolite ratios and ND outcome for preterm-born children. The relationship between ND and altered NAA/Cho specifically has been studied more extensively, still it is unclear whether an association exists. In a recent systematic review, the authors showed that NAA/Cho ratios measured at term equivalent age can serve as a potential surrogate marker for the short-term outcome.^26^ Findings of a relationship between white matter NAA/Cho ratios have been shown for motor outcome in preterm infants at 1 year of age^46–48^ as well as for cognitive and language outcome^27,46^ continuing at 18–24 months of corrected age for all developmental domains, but the relationship to longer-term outcome needs to be studied.^26^

Interestingly, metabolite ratios in preterm-born teenagers and adults remain altered and appear to be associated with cognitive function.^49,50^ Studying the relationship between metabolite ratios and ND is challenging as both parameters change throughout childhood. However, even in the absence of an association with the 1-year neurodevelopmental outcome, NAA/Cho as an expression of altered brain maturation could still be associated with more complex cognitive and language functions that only evolve later during childhood and may not be captured as early as 1 year of age. Further studies would be needed to evaluate the link between neurometabolite changes and outcome at a later developmental stage.

The underlying mechanisms of impaired ND outcome at 1 year of age are multifactorial. In this analysis, we focused solely on the impact of altered metabolic ratios, but further studies are needed to improve our understanding of the multiple pathological findings in CHD patients. For an early identification of children at risk for ND impairment, which is needed for early therapeutic support, a risk score derived from a variety of pathological MR findings as well as various clinical variables and psychosocial factors may help to stratify patients according to their risk of a negative outcome. However, further research is needed to elucidate the interplay between these various factors and their combined impact on ND.

### Limitations

The following limitations of our study merit mentioning. During our data collection, a scanner software upgrade was performed, but the effects of the scanner upgrade were investigated both by introducing a variable in our model to account for the upgrade and by performing a separate analysis on the second (larger) cohort only. We had several missing values in our metabolites because of incomplete scanning protocols due to the nature of the data collection, during natural sleep. It also has to be taken into account, that our cohort is not fully representative of the clinical population as only hemodynamically stable neonates could be scanned, while more critically ill patients might be missing in our cohort. Further limitations regarding the studied patients include their heterogeneity due to the different types of CHD and the different degrees of surgical invasiveness. While we could compare the patients to a healthy control group, the controls only underwent one scan at a time between the two times of the pre- and postoperative scans. This limits the time-corrected analysis of the metabolite rate change as a linear increase with age was assumed and a longitudinal comparison was not possible. For the outcome correlation analysis, controlling for the diagnosis was challenging, but we explored two binary categorization options, non-TGA vs TGA and moderate vs severe CHD. Differentiated comparison between CHD subtypes might be necessary, ideally in larger or more homogenous cohorts. In addition, the overall good outcome of most infants in the cohort might limit the ability to detect a relationship between altered metabolite ratios and ND outcome. Finally, the investigators were not blinded to the MR findings.

## Conclusion

We found that NAA/Cho is reduced in our CHD patients compared to the healthy control group, while other neurometabolites follow the physiological course of metabolic brain development within the first month of life compared to healthy controls. No evidence for a correlation between white matter NAA/Cho and individual BCSs at 1 year of age was found. Further investigation is needed to clarify if a link exists between altered neonatal cerebral metabolite levels and outcome at a later timepoint.

## Supplementary information

Supplementary material

## Data Availability

The datasets generated during and/or analyzed during the current study are available from the corresponding author on reasonable request.
