# Supplementary material for: Neurometabolic changes in neonates with congenital heart defects and their relation to neurodevelopmental outcome
Source: Pediatr Res. 2022 Aug 22;93(6):1642–50. doi: 10.1038/s41390-022-02253-y (PMC10172141; doi:10.1038/s41390-022-02253-y)
Supplement: Supplementary file 1 — Supplementary material [file 41390_2022_2253_MOESM1_ESM.pdf]

**Supplementary Material**

**Supplementary Figure 1: Patient flowchart**

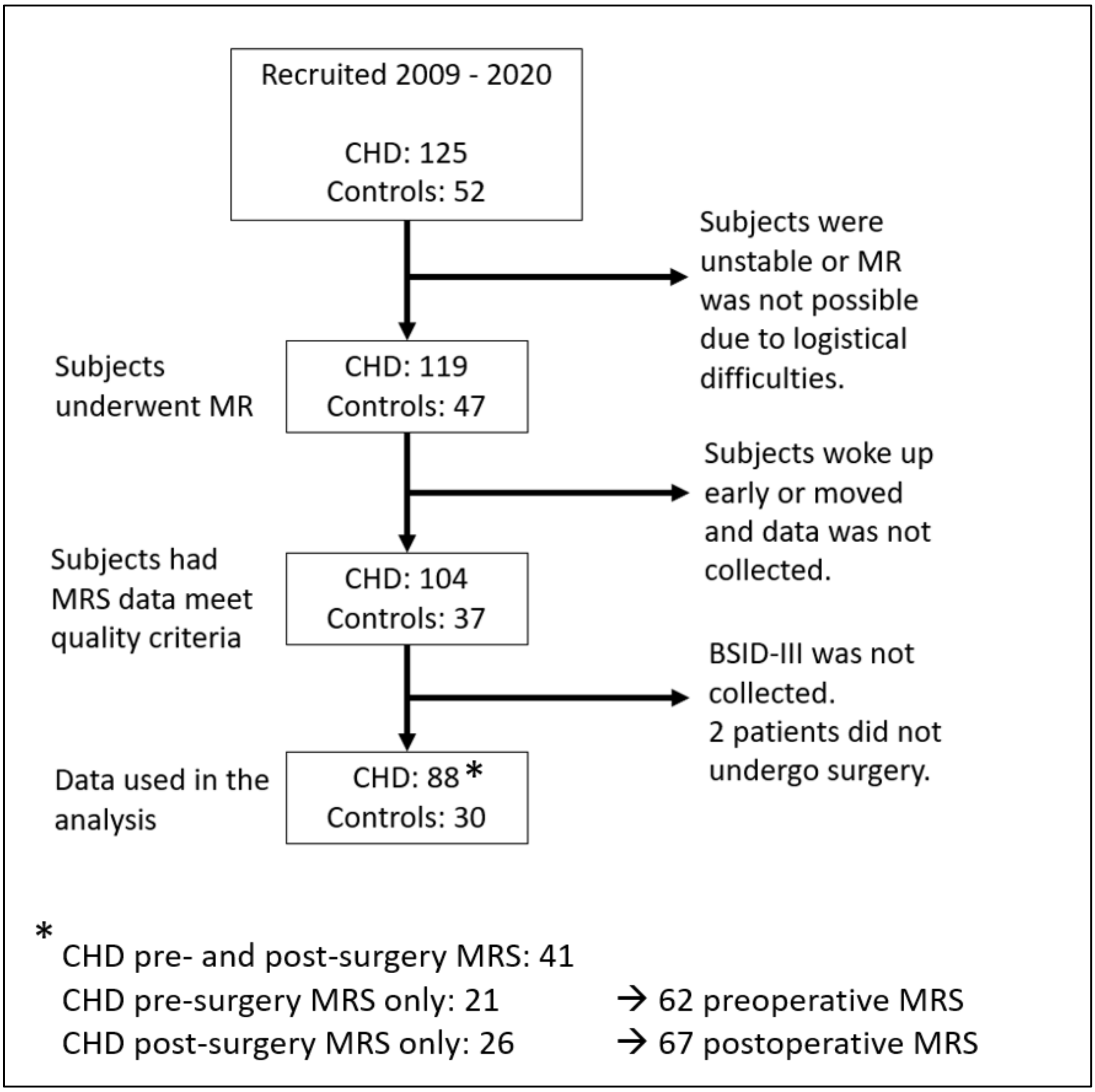

**Supplementary Table 1: Lesions**

Overview of lesions detected in the anatomical images acquired in the patients.

| | **Preoperative** | | | **Postoperative** | | |
|---|---|---|---|---|---|---|
| Abnormalisites present | | | 25 | | | 18 |
| White matter injuries | | | 11 | | | 10 |
| Affected lobes | **Left** | **Right** | **Both sides** | **Left** | **Right** | **Both sides** |
| Frontal | 1 | | | 1 | | |
| Frontal and Parietal | | | 2 | | | 2 |
| Frontal, Parietal and Temporal | | | 1 | | | |
| Parietal | 4 | 2 | 1 | 2 | 2 | 2 |
| Occipital | | | | | | |
| New lesions in-between scans (n=41) | | | | | | 6 |

**Supplementary Table 2: Metabolite differences in both cohorts**

Mixed effects linear models to analyse the effect of CHD (group variable) on metabolite ratios. bg= basal ganglia, wm= white matter, NAA= N-acetylaspartate, Cho= Choline, Cr= Creatine, MI= Myo-Inositol, Glx=glutamate and glutamine, Glu= Glutamate, Lac= Lactate. B= unstandardized regression coefficients, SE B= standard error of B, CI= confidence interval. P-values smaller than 0.05 are bold.

**bg NAA/Cho**

| term | B | SE B | CI 2.5% | CI 0.95% | p-value | FDR corrected |
|---:|---:|---:|---:|---:|---:|---:|
| (Intercept) | 2.26 | 0.08 | 2.11 | 2.41 | **>0.001** | |
| group | -0.14 | 0.07 | -0.27 | -0.01 | **0.035** | 0.175 |
| ga at mri | 0.11 | 0.01 | 0.09 | 0.13 | <**0.001** | |
| scanner software | -0.11 | 0.06 | -0.22 | 0.01 | 0.069 | |
| sex | -0.10 | 0.06 | -0.21 | 0.02 | 0.112 | |

**wm NAA/Cho**

| term | B | SE B | CI 2.5% | CI 0.95% | p-value | FDR corrected |
|---:|---:|---:|---:|---:|---:|---:|
| (Intercept) | 2.11 | 0.08 | 1.96 | 2.27 | <**0.001** | |
| group | -0.26 | 0.07 | -0.40 | -0.13 | <**0.001** | **0.010** |
| ga at mri | 0.14 | 0.01 | 0.11 | 0.17 | <**0.001** | |
| scanner software | -0.02 | 0.06 | -0.13 | 0.10 | 0.769 | |
| sex | -0.03 | 0.06 | -0.14 | 0.09 | 0.633 | |

**bg glu/Cr**

| term | B | SE B | CI 2.5% | CI 0.95% | p-value | FDR corrected |
|---:|---:|---:|---:|---:|---:|---:|
| (Intercept) | 0.94 | 0.05 | 0.84 | 1.03 | <**0.001** | |
| group | 0.02 | 0.04 | -0.06 | 0.11 | 0.571 | 0.714 |
| ga at mri | 0.02 | 0.01 | 0.00 | 0.03 | **0.040** | |
| scanner software | 0.15 | 0.04 | 0.08 | 0.22 | <**0.001** | |
| sex | -0.05 | 0.04 | -0.12 | 0.03 | 0.232 | |

**wm glu/Cr**

| term | B | SE B | CI 2.5% | CI 0.95% | p-value | FDR corrected |
|---:|---:|---:|---:|---:|---:|---:|
| (Intercept) | 1.22 | 0.08 | 1.07 | 1.37 | <**0.001** | |
| group | 0.06 | 0.07 | -0.07 | 0.18 | 0.398 | 0.651 |
| ga at mri | 0.03 | 0.01 | 0.01 | 0.06 | **0.008** | |
| scanner software | 0.21 | 0.06 | 0.10 | 0.31 | <**0.001** | |
| sex | -0.07 | 0.06 | -0.18 | 0.04 | 0.210 | |

**bg Glx/Cr**

| term | B | SE B | CI 2.5% | CI 0.95% | p-value | FDR corrected |
|---|---|---|---|---|---|---|
| (Intercept) | 1.40 | 0.10 | 1.22 | 1.59 | <**0.001** | |
| group | 0.13 | 0.08 | -0.03 | 0.29 | 0.120 | 0.244 |
| ga at mri | 0.01 | 0.02 | -0.02 | 0.04 | 0.456 | |
| scanner software | 0.28 | 0.07 | 0.15 | 0.42 | <**0.001** | |
| sex | -0.13 | 0.07 | -0.27 | 0.01 | 0.077 | |

**wm Glx/Cr**

| term | B | SE B | CI 2.5% | CI 0.95% | p-value | FDR corrected |
|---|---|---|---|---|---|---|
| (Intercept) | 1.89 | 0.13 | 1.64 | 2.15 | <**0.001** | |
| group | 0.04 | 0.11 | -0.18 | 0.26 | 0.739 | 0.779 |
| ga at mri | 0.01 | 0.02 | -0.04 | 0.05 | 0.761 | |
| scanner software | 0.40 | 0.10 | 0.21 | 0.58 | <**0.001** | |
| sex | -0.07 | 0.10 | -0.26 | 0.12 | 0.483 | |

**bg mI/Cr**

| term | B | SE B | CI 2.5% | CI 0.95% | p-value | FDR corrected |
|---|---|---|---|---|---|---|
| (Intercept) | 0.76 | 0.05 | 0.67 | 0.85 | <**0.001** | |
| group | 0.03 | 0.04 | -0.05 | 0.11 | 0.456 | 0.651 |
| ga at mri | -0.03 | 0.01 | -0.05 | -0.02 | <**0.001** | |
| scanner software | 0.03 | 0.03 | -0.04 | 0.10 | 0.383 | |
| sex | -0.02 | 0.03 | -0.09 | 0.04 | 0.488 | |

**wm mI/Cr**

| term | B | SE B | CI 2.5% | CI 0.95% | p-value | FDR corrected |
|---|---|---|---|---|---|---|
| (Intercept) | 1.43 | 0.07 | 1.29 | 1.57 | <**0.001** | |
| group | -0.02 | 0.06 | -0.13 | 0.10 | 0.799 | 0.779 |
| ga at mri | -0.06 | 0.01 | -0.09 | -0.04 | <**0.001** | |
| scanner software | -0.09 | 0.05 | -0.19 | 0.01 | 0.069 | |
| sex | 0.12 | 0.05 | 0.02 | 0.22 | **0.020** | |

**bg log(Lac/Cr)**

| term | B | SE B | CI 2.5% | CI 0.95% | p-value | FDR corrected |
|---|---|---|---|---|---|---|
| (Intercept) | -3.06 | 0.43 | -3.89 | -2.24 | <**0.001** | |
| group | 0.58 | 0.37 | -0.14 | 1.30 | 0.122 | 0.244 |
| ga at mri | -0.19 | 0.07 | -0.33 | -0.05 | **0.009** | |
| scanner software | -0.09 | 0.31 | -0.69 | 0.51 | 0.770 | |
| sex | -0.58 | 0.32 | -1.20 | 0.03 | 0.066 | |

**wm log(Lac/Cr)**

| term | B | SE B | CI 2.5% | CI 0.95% | p-value | FDR corrected |
|---|---|---|---|---|---|---|
| (Intercept) | -1.90 | 0.42 | -2.71 | -1.08 | **<0.001** | |
| group | 0.60 | 0.36 | -0.11 | 1.30 | 0.103 | 0.244 |
| ga at mri | -0.14 | 0.07 | -0.29 | 0.00 | 0.054 | |
| scanner software | -1.14 | 0.30 | -1.71 | -0.57 | **<0.001** | |
| sex | -0.02 | 0.30 | -0.60 | 0.56 | 0.948 | |

**Supplementary Table 3: Metabolite differences in second cohort only**

Mixed effects linear models to analyse the effect of CHD (group variable) on metabolite ratios. bg= basal ganglia, wm= white matter, NAA= N-acetylaspartate, Cho= Choline, Cr= Creatine, MI= Myo-Inositol, Glx=glutamate and glutamine, Glu= Glutamate, Lac= Lactate. B= unstandardized regression coefficients, SE B= standard error of B, CI= confidence interval. P-values smaller than 0.05 are bold.

**bg NAA/Cho**

| term | B | SE B | CI 2.5% | CI 0.95% | p-value |
|---:|---:|---:|---:|---:|---:|
| (Intercept) | 2.13 | 0.08 | 1.97 | 2.29 | **<0.001** |
| group | -0.10 | 0.09 | -0.27 | 0.07 | 0.260 |
| ga at mri | 0.11 | 0.01 | 0.08 | 0.14 | **<0.001** |
| sex | -0.12 | 0.08 | -0.27 | 0.03 | 0.129 |

**wm NAA/Cho**

| term | B | SE B | CI 2.5% | CI 0.95% | p-value |
|---:|---:|---:|---:|---:|---:|
| (Intercept) | 2.13 | 0.08 | 1.97 | 2.30 | **<0.001** |
| group | -0.33 | 0.09 | -0.50 | -0.17 | **<0.001** |
| ga at mri | 0.13 | 0.02 | 0.09 | 0.16 | **<0.001** |
| sex | 0.00 | 0.07 | -0.14 | 0.14 | 0.988 |

**bg glu/Cr**

| term | B | SE B | CI 2.5% | CI 0.95% | p-value |
|---:|---:|---:|---:|---:|---:|
| (Intercept) | 1.09 | 0.06 | 0.98 | 1.20 | **<0.001** |
| group | 0.01 | 0.06 | -0.10 | 0.12 | 0.893 |
| ga at mri | 0.02 | 0.01 | 0.00 | 0.04 | 0.103 |
| sex | -0.03 | 0.05 | -0.13 | 0.06 | 0.479 |

**wm glu/Cr**

| term | B | SE B | CI 2.5% | CI 0.95% | p-value |
|---:|---:|---:|---:|---:|---:|
| (Intercept) | 1.43 | 0.08 | 1.28 | 1.59 | **<0.001** |
| group | 0.05 | 0.08 | -0.11 | 0.21 | 0.536 |
| ga at mri | 0.03 | 0.02 | 0.00 | 0.06 | 0.072 |
| sex | -0.08 | 0.07 | -0.22 | 0.06 | 0.278 |

**bg Glx/Cr**

| term | B | SE B | CI 2.5% | CI 0.95% | p-value |
|---:|---:|---:|---:|---:|---:|
| (Intercept) | 1.68 | 0.11 | 1.47 | 1.90 | **<0.001** |
| group | 0.11 | 0.11 | -0.11 | 0.33 | 0.327 |
| ga at mri | 0.02 | 0.02 | -0.02 | 0.06 | 0.318 |
| sex | -0.10 | 0.09 | -0.29 | 0.08 | 0.275 |

**wm Glx/Cr**

| term | B | SE B | CI 2.5% | CI 0.95% | p-value |
|---:|---:|---:|---:|---:|---:|

| | | | | | |
|---|---|---|---|---|---|
| (Intercept) | 2.29 | 0.14 | 2.01 | 2.57 | **<0.001** |
| group | 0.00 | 0.15 | -0.29 | 0.29 | 0.994 |
| ga at mri | 0.01 | 0.03 | -0.05 | 0.06 | 0.801 |
| sex | -0.02 | 0.13 | -0.28 | 0.23 | 0.866 |

**bg mI/Cr**

| term | B | SE B | CI 2.5% | CI 0.95% | p-value |
|---|---|---|---|---|---|
| (Intercept) | 0.79 | 0.05 | 0.69 | 0.89 | **<0.001** |
| group | 0.00 | 0.05 | -0.11 | 0.10 | 0.928 |
| ga at mri | -0.03 | 0.01 | -0.05 | -0.01 | **0.002** |
| sex | 0.01 | 0.05 | -0.08 | 0.10 | 0.835 |

**wm mI/Cr**

| term | B | SE B | CI 2.5% | CI 0.95% | p-value |
|---|---|---|---|---|---|
| (Intercept) | 1.37 | 0.08 | 1.22 | 1.52 | **<0.001** |
| group | -0.05 | 0.08 | -0.20 | 0.11 | 0.559 |
| ga at mri | -0.05 | 0.02 | -0.09 | -0.02 | **0.001** |
| sex | 0.11 | 0.07 | -0.02 | 0.23 | 0.113 |

**bg log(Lac/Cr)**

| term | B | SE B | CI 2.5% | CI 0.95% | p-value |
|---|---|---|---|---|---|
| (Intercept) | -2.90 | 0.43 | -3.74 | -2.05 | **<0.001** |
| group | 0.04 | 0.45 | -0.83 | 0.91 | 0.926 |
| ga at mri | -0.24 | 0.08 | -0.40 | -0.09 | **0.004** |
| sex | -0.29 | 0.37 | -1.02 | 0.43 | 0.432 |

**wm log(Lac/Cr)**

| term | B | SE B | CI 2.5% | CI 0.95% | p-value |
|---|---|---|---|---|---|
| (Intercept) | -3.26 | 0.50 | -4.23 | -2.29 | **<0.001** |
| group | 0.83 | 0.51 | -0.16 | 1.82 | 0.106 |
| ga at mri | -0.19 | 0.10 | -0.39 | 0.01 | 0.074 |
| sex | 0.02 | 0.43 | -0.80 | 0.85 | 0.961 |

**Supplementary Table 4: white matter NAA/Cho and Outcome (diagnosis dTAG vs. non-dTGA)**

Results of linear models to analyse the relationship between white matter Naa/Cho and Bayley composite scores (CCS, LCS, MCS). NAA= N-acetylaspartate, Cho= Choline, ga= gestational age, wm= white matter, CCS = Bayley cognition composite score, LCS = Bayley language composite score, MCS = Bayley motor composite score. B= unstandardized regression coefficients, SE B= standard error of B, CI= confidence interval. P-values smaller than 0.05 are bold (uncorrected).

**Pre-operative**

**MCS** — $R^2 = 0.21$ adj. $R^2 = 0.148$

| term | B | SE | CI 2.5% | CI 97.5% | p-value |
|---|---|---|---|---|---|
| (Intercept) | -17.58 | 16.88 | -51.47 | 16.30 | 0.303 |
| SES | 1.87 | 0.98 | -0.09 | 3.84 | 0.061 |
| non-TGA | -8.65 | 4.26 | -17.19 | -0.10 | **0.047** |
| wm Naa/Cho | 4.62 | 8.54 | -12.51 | 21.76 | 0.590 |
| ga at MRI | 1.49 | 1.85 | -2.23 | 5.21 | 0.425 |

**LCS** — $R^2 = 0.16$ adj. $R^2 = 0.095$

| term | B | SE | CI 2.5% | CI 97.5% | p-value |
|---|---|---|---|---|---|
| (Intercept) | -35.83 | 15.38 | -51.47 | 16.30 | **0.024** |
| SES | 2.44 | 0.86 | -0.09 | 3.84 | **0.007** |
| non-TGA | 2.17 | 3.66 | -17.19 | -0.10 | 0.556 |
| wm Naa/Cho | 8.50 | 7.46 | -12.51 | 21.76 | 0.260 |
| ga at MRI | -0.10 | 1.59 | -2.23 | 5.21 | 0.949 |

**CCS** — $R^2 = 0.18$ adj. $R^2 = 0.112$

| term | B | SE | CI 2.5% | CI 97.5% | p-value |
|---|---|---|---|---|---|
| (Intercept) | -17.56 | 16.53 | -50.74 | 15.63 | 0.293 |
| SES | 1.84 | 0.96 | -0.09 | 3.76 | 0.061 |
| non-TGA | -5.27 | 4.17 | -13.64 | 3.10 | 0.212 |
| wm Naa/Cho | 4.44 | 8.36 | -12.51 | 21.22 | 0.598 |
| ga at MRI | 2.33 | 1.82 | -1.32 | 5.97 | 0.205 |

**Post-operative**

**MCS** — $R^2 = 0.21$ adj. $R^2 = 0.148$

| term | B | SE | CI 2.5% | CI 97.5% | p-value |
|---|---|---|---|---|---|
| (Intercept) | -21.108 | 11.993 | -45.184 | 2.968 | 0.084 |
| SES | 2.056 | 0.896 | 0.257 | 3.856 | **0.026** |
| non-TGA | -2.467 | 3.816 | -10.127 | 5.194 | 0.521 |
| wm Naa/Cho | 2.162 | 4.794 | -7.462 | 11.785 | 0.654 |
| ga at MRI | 0.759 | 1.333 | -1.918 | 3.436 | 0.572 |

**LCS** — $R^2 = 0.19$ adj. $R^2 = 0.12$

| term | B | SE | CI 2.5% | CI 97.5% | p-value |
|---|---|---|---|---|---|
| (Intercept) | -3.295 | 10.227 | -23.858 | 17.269 | 0.749 |
| SES | 1.278 | 0.749 | -0.228 | 2.785 | 0.095 |
| non-TGA | 4.878 | 3.123 | -1.401 | 11.157 | 0.125 |
| wm Naa/Cho | -6.453 | 3.877 | -14.248 | 1.343 | 0.103 |
| ga at MRI | 2.594 | 1.061 | 0.461 | 4.726 | **0.018** |

**CCS** — $R^2 = 0.14$ adj. $R^2 = 0.079$

| term | B | SE | CI 2.5% | CI 97.5% | p-value |
|---|---|---|---|---|---|
| (Intercept) | -6.795 | 11.409 | -29.699 | 16.109 | 0.554 |
| SES | 1.785 | 0.853 | 0.073 | 3.497 | **0.041** |
| non-TGA | -4.866 | 3.630 | -12.154 | 2.422 | 0.186 |
| wm Naa/Cho | -4.208 | 4.560 | -13.363 | 4.947 | 0.361 |
| ga at MRI | 1.539 | 1.269 | -1.008 | 4.085 | 0.231 |

**Supplementary Table 5: white matter NAA/Cho and Outcome corrected for white matter injuries (diagnosis dTGA vs. non-dTGA)**

Results of linear models to analyse the relationship between white matter Naa/Cho and Bayley composite scores (CCS, LCS, MCS). NAA= N-acetylaspartate, Cho= Choline, ga= gestational age, wm= white matter, CCS = Bayley cognition composite score, LCS = Bayley language composite score, MCS = Bayley motor composite score, WMI= white matter injury. B= unstandardized regression coefficients, SE B= standard error of B, CI= confidence interval. P-values smaller than 0.05 are bold (uncorrected).

**Pre-operative**

| **MCS** | | | | | $R^2 = 0.21$ adj. $R^2 = 0.13$ |
|---|---|---|---|---|---|
| term | B | SE | CI 2.5% | CI 97.5% | p-value |
| (Intercept) | -18.04 | 17.05 | -52.29 | 16.20 | 0.30 |
| SES | 1.93 | 1.00 | -0.07 | 3.93 | 0.06 |
| non-TGA | -8.67 | 4.29 | -17.28 | -0.05 | **0.049** |
| wm Naa/Cho | 4.79 | 8.61 | -12.51 | 22.09 | 0.58 |
| ga at MRI | 1.44 | 1.87 | -2.32 | 5.20 | 0.46 |
| WMI present | -2.32 | 5.40 | -13.16 | 8.53 | 0.67 |

| **LCS** | | | | | $R^2 = 0.16$ adj. $R^2 = 0.08$ |
|---|---|---|---|---|---|
| term | B | SE | CI 2.5% | CI 97.5% | p-value |
| (Intercept) | -35.73 | 15.56 | -67.00 | -4.47 | **0.03** |
| SES | 2.43 | 0.89 | 0.66 | 4.19 | **0.01** |
| non-TGA | 2.17 | 3.70 | -5.26 | 9.60 | 0.56 |
| wm Naa/Cho | 8.47 | 7.54 | -6.67 | 23.62 | 0.27 |
| ga at MRI | -0.09 | 1.61 | -3.33 | 3.15 | 0.95 |
| WMI present | 0.54 | 4.60 | -8.70 | 9.79 | 0.91 |

| **CCS** | | | | | $R^2 = 0.18$ adj. $R^2 = 0.10$ |
|---|---|---|---|---|---|
| term | B | SE | CI 2.5% | CI 97.5% | p-value |
| (Intercept) | -17.69 | 16.73 | -51.28 | 15.91 | 0.30 |
| SES | 1.85 | 0.98 | -0.11 | 3.82 | 0.06 |
| non-TGA | -5.27 | 4.21 | -13.73 | 3.18 | 0.22 |
| wm Naa/Cho | 4.49 | 8.45 | -12.49 | 21.46 | 0.60 |
| ga at MRI | 2.31 | 1.84 | -1.38 | 6.00 | 0.21 |
| WMI present | -0.67 | 5.30 | -11.31 | 9.97 | 0.90 |

**Post-operative**

**MCS** — $R^2$ = 0.15 adj. $R^2$ =0.07

| term | B | SE | CI 2.5% | CI 97.5% | p-value |
|---|---|---|---|---|---|
| (Intercept) | -20.65 | 12.02 | -44.80 | 3.50 | 0.09 |
| SES | 2.08 | 0.90 | 0.28 | 3.88 | **0.025** |
| non-TGA | -3.33 | 3.94 | -11.23 | 4.58 | 0.40 |
| wm Naa/Cho | 2.47 | 4.81 | -7.20 | 12.14 | 0.61 |
| ga at MRI | 0.53 | 1.36 | -2.19 | 3.26 | 0.70 |
| WMI present | -5.01 | 5.49 | -16.03 | 6.01 | 0.37 |

**LCS** — $R^2$ =0.26 adj. $R^2$ =0.18

| term | B | SE | CI 2.5% | CI 97.5% | p-value |
|---|---|---|---|---|---|
| (Intercept) | -2.07 | 9.91 | -22.00 | 17.87 | 0.84 |
| SES | 1.30 | 0.72 | -0.16 | 2.75 | 0.08 |
| non-TGA | 3.31 | 3.11 | -2.95 | 9.58 | 0.29 |
| wm Naa/Cho | -6.00 | 3.76 | -13.56 | 1.56 | 0.12 |
| ga at MRI | 2.22 | 1.04 | 0.12 | 4.31 | **0.04** |
| WMI present | -8.66 | 4.17 | -17.04 | -0.28 | **0.04** |

**CCS** — $R^2$ =0.15 adj. $R^2$ =0.06

| term | B | SE | CI 2.5% | CI 97.5% | p-value |
|---|---|---|---|---|---|
| (Intercept) | -6.98 | 11.52 | -30.11 | 16.15 | 0.55 |
| SES | 1.76 | 0.86 | 0.05 | 3.50 | **0.04** |
| non-TGA | -4.52 | 3.77 | -12.10 | 3.05 | 0.24 |
| wm Naa/Cho | -4.33 | 4.61 | -13.59 | 4.93 | 0.35 |
| ga at MRI | 1.62 | 1.30 | -0.98 | 4.24 | 0.22 |
| WMI present | 1.99 | 5.26 | -8.47 | 12.54 | 0.71 |

**Supplementary Table 6: white matter NAA/Cho and Outcome (diagnosis moderate vs. severe)**

Results of linear models to analyse the relationship between white matter Naa/Cho and Bayley composite scores (CCS, LCS, MCS). NAA= N-acetylaspartate, Cho= Choline, ga= gestational age, wm= white matter, CCS = Bayley cognition composite score, LCS = Bayley language composite score, MCS = Bayley motor composite score. B= unstandardized regression coefficients, SE B= standard error of B, CI= confidence interval. P-values smaller than 0.05 are bold (uncorrected).

**Pre-operative**

| **MCS** | | | | | $R^2$ = 0.33 adj. $R^2$ =0.27 |
|---|---|---|---|---|---|
| term | B | SE | CI 2.5% | CI 97.5% | p-value |
| (Intercept) | -27.33 | 15.55 | -58.55 | 3.89 | 0.8 |
| SES | 1.86 | 0.88 | 0.09 | 3.62 | **0.04** |
| Severe CHD | -16.31 | 4.41 | -25.16 | -7.46 | < **0.001** |
| wm Naa/Cho | 10.38 | 8.10 | -5.88 | 26.65 | 0.21 |
| ga at MRI | 0.40 | 1.75 | -3.16 | 3.91 | 0.82 |

| **LCS** | | | | | $R^2$ =0.16 adj. $R^2$ =0.09 |
|---|---|---|---|---|---|
| term | B | SE | CI 2.5% | CI 97.5% | p-value |
| (Intercept) | -34.32 | 15.27 | -65.00 | -3.65 | **0.03** |
| SES | 2.28 | 0.84 | 0.60 | 3.96 | **0.009** |
| Severe CHD | 0.06 | 4.08 | -8.13 | 8.26 | 0.99 |
| wm Naa/Cho | 8.78 | 7.69 | -6.66 | 24.23 | 0.26 |
| ga at MRI | -0.26 | 1.64 | -3.54 | 3.03 | 0.88 |

| **CCS** | | | | | $R^2$ =0.17 adj. $R^2$ =0.10 |
|---|---|---|---|---|---|
| term | B | SE | CI 2.5% | CI 97.5% | p-value |
| (Intercept) | -21.41 | 16.64 | -54.81 | 12.00 | 0.20 |
| SES | 2.04 | 0.94 | 0.15 | 3.93 | **0.03** |
| Severe CHD | -3.99 | 4.72 | -13.46 | 5.49 | 0.40 |
| wm Naa/Cho | 5.14 | 8.67 | -12.26 | 22.55 | 0.55 |
| ga at MRI | 2.33 | 1.87 | -1.43 | 6.09 | 0.22 |

**Post-operative**

**MCS** $R^2 = 0.21$ adj. $R^2 =0.15$

| term | B | SE | CI 2.5% | CI 97.5% | p-value |
|---|---|---|---|---|---|
| (Intercept) | -17.04 | 11.21 | -39.55 | 5.46 | 0.13 |
| SES | 1.86 | 0.85 | 0.15 | 3.58 | **0.03** |
| Severe CHD | -9.52 | 4.12 | -17.78 | -1.26 | **0.02** |
| wm Naa/Cho | 1.71 | 4.57 | -7.45 | 10.88 | 0.71 |
| ga at MRI | 0.66 | 1.27 | -1.90 | 3.22 | 0.61 |

**LCS** $R^2 =0.17$ adj. $R^2 =0.10$

| term | B | SE | CI 2.5% | CI 97.5% | p-value |
|---|---|---|---|---|---|
| (Intercept) | -0.53 | 10.07 | -20.78 | 19.71 | 0.96 |
| SES | 1.18 | 0.75 | -0.33 | 2.70 | 0.12 |
| Severe CHD | 3.87 | 3.67 | -3.50 | 11.24 | 0.30 |
| wm Naa/Cho | -6.89 | 3.91 | -14.75 | 0.97 | 0.08 |
| ga at MRI | 2.61 | 1.07 | 0.44 | 4.77 | **0.02** |

**CCS** $R^2 =0.12$ adj. $R^2 =0.05$

| term | B | SE | CI 2.5% | CI 97.5% | p-value |
|---|---|---|---|---|---|
| (Intercept) | -10.12 | 11.32 | -32.85 | 12.62 | 0.38 |
| SES | 1.89 | 0.86 | 0.16 | 3.63 | **0.03** |
| Severe CHD | -2.33 | 4.16 | -10.68 | 6.02 | 0.58 |
| wm Naa/Cho | -3.74 | 4.61 | -13.00 | 5.52 | 0.42 |
| ga at MRI | 1.56 | 1.29 | -1.02 | 4.15 | 0.23 |

**Supplementary Table 7: white matter NAA/Cho and Outcome corrected for white matter injuries (diagnosis moderate vs. severe)**

Results of linear models to analyse the relationship between white matter Naa/Cho and Bayley composite scores (CCS, LCS, MCS). NAA= N-acetylaspartate, Cho= Choline, ga= gestational age, wm= white matter, CCS = Bayley cognition composite score, LCS = Bayley language composite score, MCS = Bayley motor composite score. B= unstandardized regression coefficients, SE B= standard error of B, CI= confidence interval. P-values smaller than 0.05 are bold (uncorrected).

**Pre-operative**

| **MCS** | | | | | $R^2 = 0.34$ adj. $R^2 = 0.27$ |
|---|---|---|---|---|---|
| term | B | SE | CI 2.5% | CI 97.5% | p-value |
| (Intercept) | -28.57 | 15.60 | -59.90 | 2.77 | 0.07 |
| SES | 1.96 | 0.89 | 0.18 | 3.74 | **0.03** |
| Severe CHD | -16.97 | 4.46 | -25.92 | -8.01 | **<0.001** |
| wm Naa/Cho | 11.04 | 8.13 | -5.29 | 27.37 | 0.18 |
| ga at MRI | 0.22 | 1.76 | -3.32 | 3.75 | 0.90 |
| WMI present | -5.00 | 5.00 | -15.05 | 5.04 | 0.32 |

| **LCS** | | | | | $R^2 = 0.16$ adj. $R^2 = 0.07$ |
|---|---|---|---|---|---|
| term | B | SE | CI 2.5% | CI 97.5% | p-value |
| (Intercept) | -34.21 | 15.46 | -65.28 | -3.13 | **0.03** |
| SES | 2.27 | 0.85 | 0.56 | 3.98 | **0.01** |
| Severe CHD | 0.13 | 4.17 | -8.24 | 8.51 | 0.97 |
| wm Naa/Cho | 8.72 | 7.79 | -6.93 | 24.37 | 0.27 |
| ga at MRI | -0.24 | 1.66 | -3.58 | 3.10 | 0.89 |
| WMI present | 0.53 | 4.67 | -8.85 | 9.91 | 0.90 |

| **CCS** | | | | | $R^2 = 0.17$ adj. $R^2 = 0.09$ |
|---|---|---|---|---|---|
| term | B | SE | CI 2.5% | CI 97.5% | p-value |
| (Intercept) | -21.72 | 16.85 | -55.57 | 12.12 | 0.20 |
| SES | 2.07 | 0.96 | 0.15 | 3.99 | **0.04** |
| Severe CHD | -4.16 | 4.81 | -13.83 | 5.51 | 0.39 |
| wm Naa/Cho | 5.31 | 8.78 | -12.32 | 22.95 | 0.55 |
| ga at MRI | 2.28 | 1.90 | -1.53 | 6.10 | 0.24 |
| WMI present | -1.29 | 5.40 | -12.13 | 9.56 | 0.81 |

**Post-operative**

| **MCS** | | | | | $R^2$ = 0.23 adj. $R^2$ =0.15 |
|---|---|---|---|---|---|
| term | B | SE | CI 2.5% | CI 97.5% | p-value |
| (Intercept) | -17.13 | 11.20 | -39.62 | 5.37 | 0.13 |
| SES | 1.91 | 0.85 | 0.19 | 3.62 | **0.03** |
| Severe CHD | -10.03 | 4.14 | -18.35 | -1.71 | **0.02** |
| wm Naa/Cho | 2.23 | 4.58 | -7.07 | 11.32 | 0.64 |
| ga at MRI | 0.42 | 1.29 | -2.18 | 3.02 | 0.75 |
| WMI present | -5.35 | 5.11 | -15.62 | 4.91 | 0.30 |

| **LCS** | | | | | $R^2$ =0.25 adj. $R^2$ =0.17 |
|---|---|---|---|---|---|
| term | B | SE | CI 2.5% | CI 97.5% | p-value |
| (Intercept) | -0.47 | 9.65 | -19.88 | 18.95 | 0.96 |
| SES | 1.24 | 0.72 | -0.21 | 2.70 | **0.09** |
| Severe CHD | 2.97 | 3.54 | -4.14 | 10.08 | 0.41 |
| wm Naa/Cho | -6.20 | 3.76 | -13.76 | 1.37 | 0.11 |
| ga at MRI | 2.21 | 1.05 | 0.10 | 4.31 | **0.04** |
| WMI present | -9.35 | 4.08 | -17.57 | -1.13 | **0.03** |

| **CCS** | | | | | $R^2$ =0.13 adj. $R^2$ =0.04 |
|---|---|---|---|---|---|
| term | B | SE | CI 2.5% | CI 97.5% | p-value |
| (Intercept) | -10.07 | 11.39 | -32.95 | 12.82 | 0.38 |
| SES | 1.87 | 0.87 | 0.12 | 3.61 | **0.04** |
| Severe CHD | -2.02 | 4.21 | -10.48 | 6.44 | 0.63 |
| wm Naa/Cho | -3.99 | 4.66 | -13.35 | 5.36 | 0.40 |
| ga at MRI | 1.71 | 1.32 | -0.94 | 4.35 | 0.20 |
| WMI present | 3.20 | 5.20 | -7.24 | 13.65 | 0.54 |
